# Handy-Book of the Treatment of Women’s and Children’s Diseases

**Published:** 1871-11

**Authors:** 


*Handy-Book of the Treatment of Women's and Children's Diseases, according to the Vienna Medical School, with Prescriptions.* By Emil Dillnberger. Translated by Patrick Nicol, M.B. Philadelphia: Lindsay & Blakston, 1871.

The names of the various maladies of women and children are given in this work, with the briefest notice of their character. To this is added the longest and most complete set of formulas, and lists of medicines applicable to all stages and forms of the disease. It is truly the Vienna School of Medication, and wholly unsafe in the hands of young American physicians.

